# “Ab Interno” Surgery of the Schlemm’s Canal in Postuveitic Glaucoma Patients

**DOI:** 10.3390/jpm13030456

**Published:** 2023-02-28

**Authors:** Ivan Tanev, Radina Kirkova

**Affiliations:** 1 Eye Clinic “Zrenieto”, 1000 Sofia, Bulgaria; 2 Department of Ophthalmology, ENT and Maxillofacial Surgery, Medical University—Pleven, 5800 Pleven, Bulgaria; 3 Department of Ophthalmology, IRCCS HUMANITAS Research Hospital, 20089 Rozzano, Italy; 4 University First Multiprofile Hospital for Active Treatment “St Joan Krastitel”, 1000 Sofia, Bulgaria

**Keywords:** glaucoma surgery, endoscope, Schlemm‘s canal, secondary glaucoma

## Abstract

Background: Patients with uveitis have a higher risk of developing glaucoma not only because of the disease by itself, but also because of the ongoing corticosteroid therapy. The pathogenesis of uveitic glaucoma is characterized by a complex of biochemical and cellular processes, as well as morphological changes in the trabecular meshwork. Treatment of secondary chronic glaucoma is challenging and often requires different approaches and solutions. Purpose: To present the “ab interno” procedure of the Schlemm‘s canal (SC) performed with the help of TrabEx+ (MST, Redmond, WA, USA) in postuveitic glaucoma patients. Methods: The observed group included patients with postuveitic secondary glaucoma, which consisted of 12 pseudophakic patients (12 eyes). Patients are with intraocular pressure higher than 25 mmHg and on maximal local therapy. Due to insufficient conservative control on IOP, surgical solutions are needed. We describe a new, minimally invasive surgical procedure–the technique of the operation, the postoperative results and complications. Results: We present the achieved intraocular pressure (on the first day, 2 weeks, 1 month, 6, 8 12 and 18 months). The morphology of the trabecular area was demonstrated by gonioscopy. The effects of the therapy on the glaucoma progression, was evaluated with perimetry and OCT. Discussion: The following procedure is a minimally invasive procedure and provides good control of intraocular pressure. Aqueous veins in the conjunctiva are significantly preserved compared to conventional filtration trabeculectomy. This potentially modulates the physiological outflow pathways by ablating trabeculum strip the cause of increased outflow resistance-the inner wall of the SC. Conclusion: Removal of the inner wall of the SC, using Trabex+ (MST, Redmond, WA, USA), demonstrates promising results in the selected patients with a minimally invasive nature.

## 1. Introduction

Nearly 10% of patients with uveitis develop irreversible vision loss due to damage of the retina and the development of glaucoma [1,2]. Uveitic glaucoma is defined as increased intraocular pressure in patients with uveitis, with typical glaucomatous damage to the optic disc and visual field [3,4]. Patients with uveitis have a high risk of developing glaucoma not only because of the disease by itself, but also because of the ongoing corticosteroid therapy. The pathophysiological mechanisms of uveitis vary according to the various causes of the disease. For example, acute anterior uveitis of herpetic etiology, toxoplasmosis, or Posner-Schlossman syndrome is characterized by a sharp increase in the intraocular pressure, while other types of uveitis (HLA-B27, associated anterior uveitis) demonstrate a chronic increase in pressure. The pathogenesis of uveitic glaucoma is characterized by a complex of biochemical and cellular processes, as well as morphological changes in the trabecular meshwork. Treatment of secondary chronic glaucoma is challenging and often requires different approaches and solutions.

The surgical technique of incising trabecular meshwork was first described in 1936 [5] to create a continuous opening from the anterior chamber to the Canal of Schlemm. Over recent years the modification of the procedure is transformed from childhood to adults glaucomas [6,7,8,9]. Multiple variations of goniotomy are popular to lower intraocular pressure (IOP) in eyes with various forms of glaucoma; these include incisional goniotomy using a Trabectome^®^ device (NeoMedix , Tustin, CA, USA), Kahook Dual Blade^®^ device (KDB, New World Medical Inc, Rancho Cucamonga, CA, USA) or the TrabEx™/TrabEx+™ device (MicroSurgical Technology, Redmond, WA,USA), 360° trabeculotomy (gonioscopy-assisted transluminal trabeculotomy - GATT) using a suture or the iTrack^®^ device (iScience Interventional Corp, Menlo Park, CA, USA) or the TRAB^®^360/OMNI^®^ system Sight Sciences Inc., Menlo Park, CA, USA).

## 2. Purpose

To present the “ab interno” procedure of the Schlemm’s canal (SC) under endoscopic control (EndoOptiks,BVI International, Little Silver, NJ, USA) performed with the help of TrabEx+ device (MicroSurgical Technology, Redmond, WA,USA) postuveitic glaucoma patients.

## 3. Material and Methods

The observed group included HLA B27 positive, non-infectious and open-angle hypertensive patients with no recurrence of anterior uveitis for more than 8 months. The observation period is more than 18 months. It consists of 12 pseudophakic patients (6 eyes)–6 men and 6 women. Cataract surgery was performed more than 6 months before TrabEx+ − intervention. The patients were on maximum local therapy and intraocular pressure higher than 25 mmHg, presented in Table 1. The severity of glaucoma has been graded as moderate, according to ICD-9 and ICD-10, as it follows optic nerve abnormalities consistent with glaucoma and glaucomatous visual field abnormalities in one hemifield, and not within 5 degrees of fixation. 

The average age was 65.7 ± 5.5 years. with the mean intraocular pressure of 30.8 ± 4.4 mmHg. Due to the small observed group, statistical processing does not confirm or reject general conclusions. The main purpose of the study is to present the modified procedure of goniectomy under endoscopic control. 

**Figure 1:**
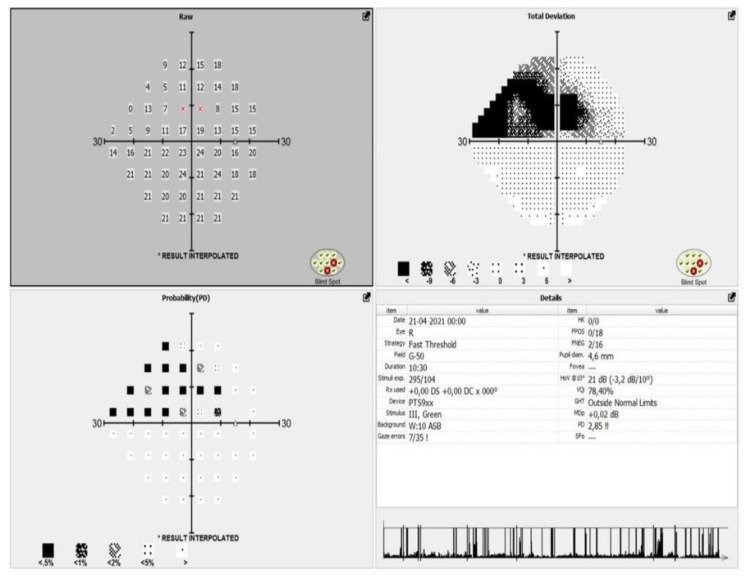
Represents the visual field of a patient with moderate glaucoma before the surgical procedure.

**Figure 2:**
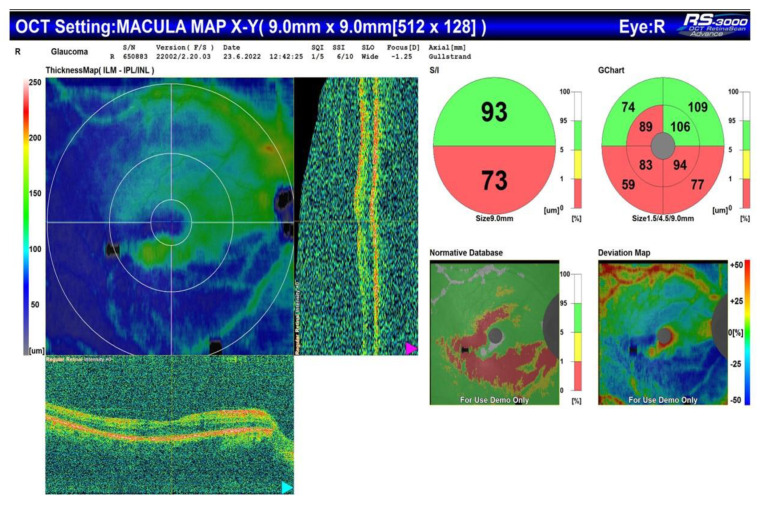
OCT of the same patient with corresponding arquate defect in the inferior half of the visual field.

All the patients included in the study meet the criteria of European glaucoma society (EGS) for moderate glaucoma, according to their visual fields and optical coherence tomography (OCT) scans (Figure 1 and Figure 2).

## 4. Surgical Procedure

After topical anaesthesia, corneal openings are made at 90° from each other, with a calibrated 1 mm diamond knife. TrabEx+ (MST, Redmond, WA, USA) is a tool for minimally invasive glaucoma surgery. It is equipped with a striated double blade that is designed to simultaneously lift and peel the trabecular meshwork. The procedure is applied at 180° in the lower nasal part of the trabeculum under direct endoscopic control. The handpiece is equipped with an irrigation-aspiration system that adapts as standard to each phacoemulsification system. Visual control and monitoring of the procedure is performed using a 23G microendoscope system (Endo Optiks, BVI Medical, Waltham, MA, USA). The result is a reduction in trabecular resistance, direct access to the collecting ducts, and increased physiological outflow through the Schlemm’s canal.

**Figure 3:**
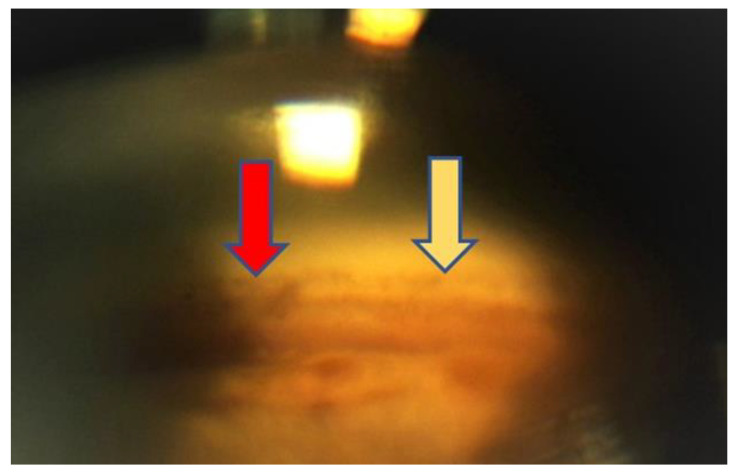
Gonioscopy view at 12 months. The area of ablation (yellow arrow) and the area of intact trabeculum (red arrow) are clearly visible.

**Figure 4:**
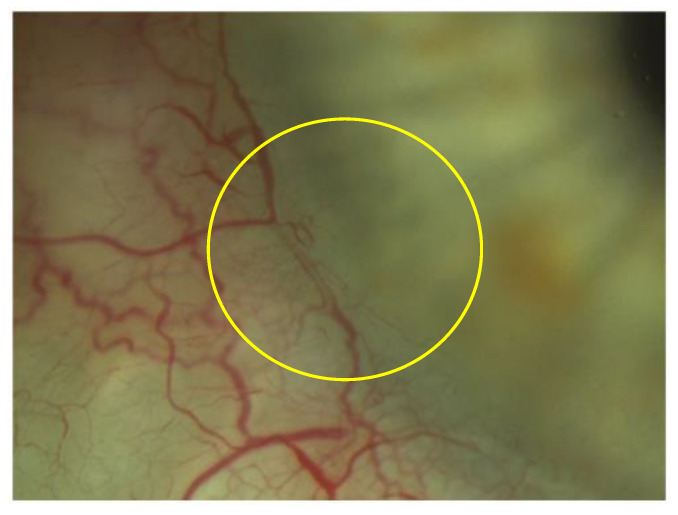
Visualization of the aqueous veins 12 months after surgery (yellow circle).

TrabEx+ (MST, Redmond, WA, USA) offers a handpiece equipped with an irrigation and aspiration system that adapts to each machine for phacoemulsification. The blade is double striated with a comfortable rounded back to form a continuous trabecular strip. When the goniectomy is performed as a strip, not just a trabeculum tear, there is a less chance of occlusion of the collector channels by the tissue. It can be seen clearly on gonioscopic view (Figure 3). Irrigation goniectomy provides consistently good visibility of the work area, and aspiration evacuates the ablated tissue. The observation and control of the procedure is carried out with an endoscope, which does not require the constant repositioning of the microscope and the patient. Months after the procedure the aqueous veins look like they are working and dilated (Figure 4). 

## 5. Results

We defined as a criterion of success of the procedure an intraocular pressure (IOP) reduction of 20% from the base line at the 18th months.

No significant intraoperative complications were observed. On the first postoperative day, microhyphema or just an insignificant amount of haematin was observed in all patients. This phenomenon is observed in all procedures based on “ab interno” surgery [10]. The measured decrease in intraocular pressure was 2 to 4 mmHg. At subsequent follow-up visits, a steady and sustained decrease in values was observed. Due to the moderate stage of the glaucomatous process and the data on improved perfusion of the optic disc by dorzolamide, it was decided to keep this medication [11,12,13].

**Figure 5:**
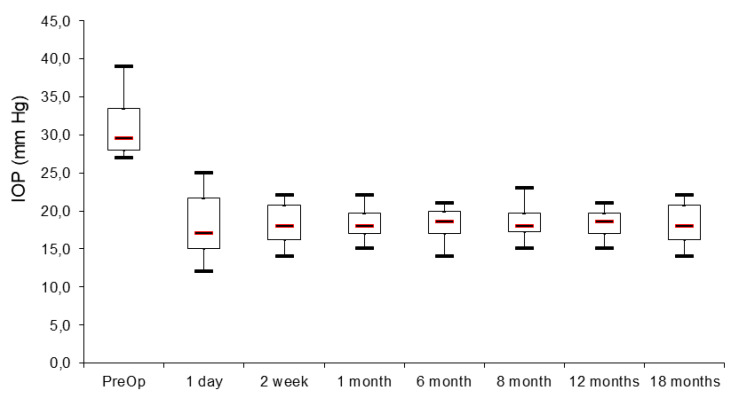
Axis “x” time, axis “y” IOP (mmHg).

Figure 5 represents the preoperative intraocular pressure with maximal local therapy (timolol 0.5% 2×, dorzolamide 2× and brimonidine 2×). The local therapy was equal for all the patients included in the study. It shows also the achieved intraocular pressure (on the first day, 2 weeks, 1 month, 6, 8, 12 and 18 months).

Figure 5 Scatterplot presents achieved intraocular pressure at 18 months post-intervention with administration of only one local medication for each patient (1–12).

**Figure 6:**
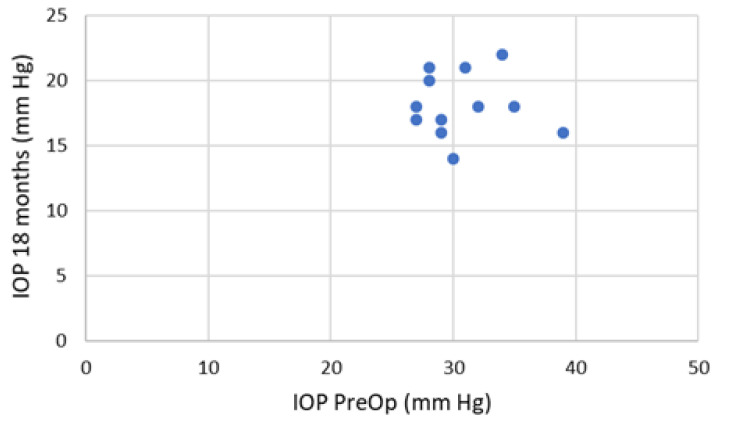
Box plot presentation of preoperative and postoperative intraocular pressure in mmHg.

**Figure 7:**
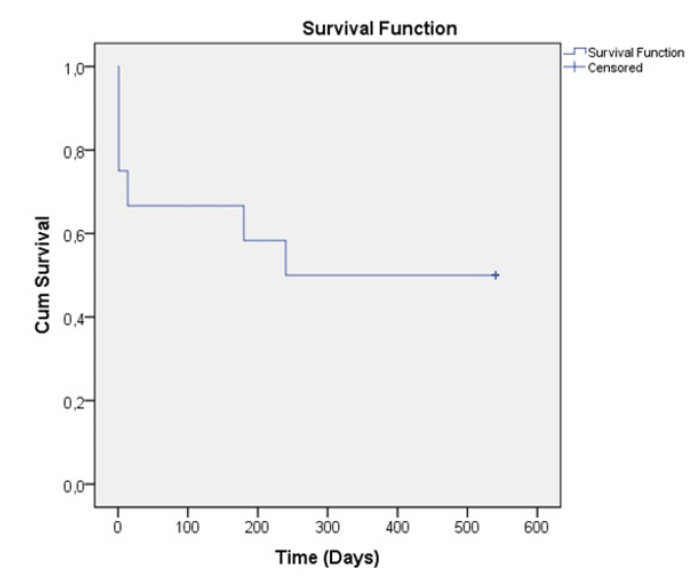
Kaplan-Meier survival estimates graph for treatment “success” at latest follow-up (intraocular pressure < 21 mm Hg; intraocular pressure lowering > 20%; no reoperation). The axis “x” shows time and the axis “y” (“cum survival”) shows the IOP after the intervention. As it can be seen from the graph, the IOP remains stable over time.

All observed patients achieved 20% and more during the follow-up period. If we look for the IOP below 21 mmHg we have 3 patients with such achievement (Figure 6). The postoperative results showed stability over time–the treatment “success” rate is represented on Figure 7, using Kaplan-Meier graph.

**Figure 8:**
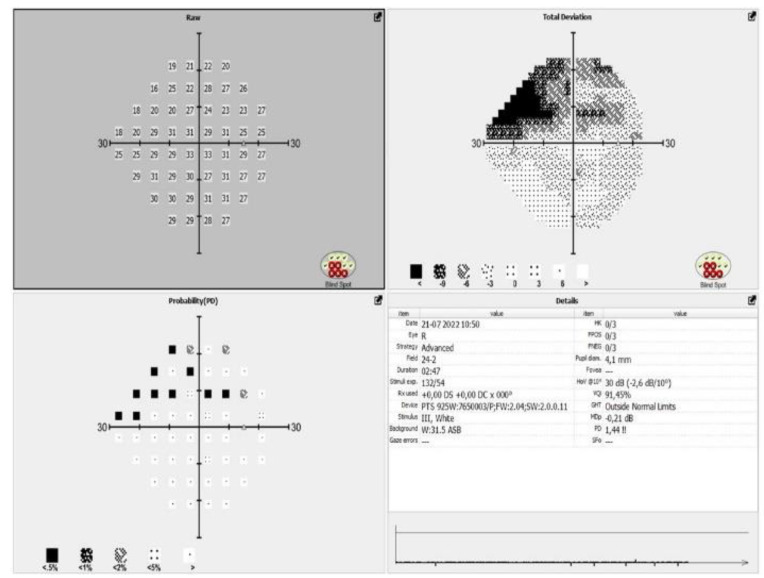
Visual field after the surgical procedure.

**Figure 9:**
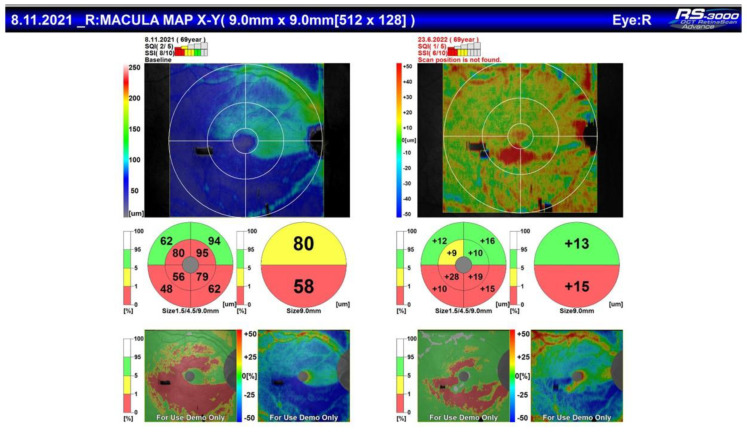
Comparison of OCT before and after the surgical procedure.

The main objective of this study is to demonstrate the effectiveness of the “ab interno” surgery of the Sclemm’s canal wall into lowering IOP in patients with moderate glaucoma, refractive to maximal treatment. However, in the obligatory diagnostic and follow-up process of this patients, all of them (like mention above), underwent OCT and visual field testing with automated perimetry. We demonstrated that no progression of visual field damage Figure 8) and no significant change in the status of RNFL (Figure 9) has been observed. 

## 6. Discussion

In recent years, glaucoma surgery has dramatically expanded the armamentarium of the available procedures. They became more selective and precise to the pathology. Other surgical techniques previously proposed for this type of glaucoma, such as deep sclerectomy or ExPRESS mini-implant have gone from being preferred to being part of a range of possible glaucoma procedures. 

Our surgery is classified in the group of ab interno Shclemm’s canal-based procedure. The proposed technique is minimally invasive and provides good control of intraocular pressure. Aqueous veins in the conjunctiva are significantly preserved, compared with conventional filtering trabeculectomy. This potentially modulates physiological outflow pathways by removing the primary cause of increased outflow resistance–the wall of the Schlemm’s canal, therebyexposing the collecting ducts. Historically, goniectomy dates back to Barkan, 1938 [14]. Initially, a cystotome was used through a single corneal incision. The main reason for failure of the operation is that the trabuculum tears and tissue are difficult to remove. Residual tissue occludes the collecting ducts again.The trabeculum removal procedure is known by several popular modern techniques such as Trabectome^®^ (Neomedix Corp), Kahook Dual Blade (KDB, New World Medical) and TrabEx (MST, Redmond). 

For the first time in 2004, Trabectome^®^ offered irrigation when opening the trabecular apparatus through a tip that forms plasma and vaporizes the tissue. In this way, direct access is provided for draining the intraocular fluid to the Schlemm’s canal and the intrascleral venous plexuses [15,16]. In 2016 Kaplowitz et al. [13] published results of a two-year study that indicated a 36% reduction in baseline intraocular pressure and a significant reduction in topical therapy to one agent.

Kahook Dual Blade (KDB, New World Medical) is a technique for trabecular band removal, using a double-edged blade positioned at an angle. The stability of the anterior chamber is maintained with an adaptive viscoelastic substance. Ammar et al. [14] demonstrated that the KDB Glide device offers reliable excision of TM, with the other devices producing incision or variable excision of tissue.

TrabEx (MST, Redmond) has the same strategy, with the blade being double striated. This allows trabeculae of varying elasticity to be ablated, reducing the frequency of band tears. The stable anterior chamber also relies on an adaptive viscoelastic substance. Gosling [15] reported in a study that irrigating goniectomy with the TrabEx+ device in combination with, or without cataract surgery, effectively decreased IOP and glaucoma medications in the postoperative period between 3 months to 2 years.

The difference between TrabEx+ and TrabEx (MST, Redmond, USA) is the handle of the TrabEx+ equipped with an irrigation and aspiration system that is adapted to each phacoemulsification machine. The application of irrigation during the procedure allows control of the reflux of blood through the collecting ducts by changing the height of the irrigation line. Ramjiani et al. [16] describe a series of cases which underwent TrabEx+ and the related histopathological findings. The primary outcome of this research was assessment of the preservation of the TM architecture after sampling.

All described techniques require observation of surgical gonio-lens. The operating microscope should be tilted approx. 40°, also the patient’s head tilts in the opposite direction. The main disadvantages of viscosubstance application methods are related to blood reflux, which necessitates frequent repositioning of the microscope and the patient’s head.

Our modification uses endoscope to observe and control the procedure. The endoscope allows to maintain the standard position of the microscope and to keep the position of the patient’s head free from tilting during any visco injection or other surgical gesture.

Considering the small sample size and absent control group are a limitation in this in this study.

## 7. Conclusions

Removal of the internal wall of the Schlemm’s canal with Trabex+ (MST, Redmond, USA), is a minimally invasive surgical procedure that demonstrated promising results in lowering IOP in postuveitic patients, with moderate form of glaucoma, according to ICD-9 and ICD-10. The achieved postoperative IOP demonstrated stability over time. Limitation of this study is the small amount of patients included and the relatively sho . It is necessary to extend the follow-up time in larger series.

## Figures and Tables

**Table 1:** Intraocular pressure (IOP) before surgery.

Patient	Sex	Age	Intraocular Pressure	Glaucoma Stage
1	Male	62	27 mmHg	moderate
2	Male	63	28 mmHg	Moderate
3	Male	58	32 mmHg	Moderate
4	Male	72	29 mmHg	Moderate
5	Female	70	30 mmHg	Moderate
6	Female	67	34 mmHg	Moderate
7	Male	68	28 mmHg	Moderate
8	Female	67	27 mmHg	Moderate
9	Female	70	29 mmHg	Moderate
10	Female	72	31 mmHg	Moderate
11	Male	61	35 mmHg	Moderate
12	Female	69	39 mmHg	Moderate

## Data Availability

All the data and materials are saved in the archives of Eye Clinic “Zrenieto”: Sofia, Bulgaria. The information about patient’s demographic characteristics, IOP measurements (as a comment in each patient’s folder) and OCT-A scans are saved in Nidek RS 3000 Advance (software version NAVIS-EX 1.8.0.) in Eye Clinic “Zrenieto”, Sofia, Bulgaria and in the archive of the Clinic.
